# Macrophages Fuel Skeletal Muscle Regeneration

**DOI:** 10.20900/immunometab20210013

**Published:** 2021-02-19

**Authors:** Joel D. Schilling

**Affiliations:** 1 Center for Cardiovascular Research, Washington University School of Medicine, St. Louis, MO 63110, USA;; 2 Department of Medicine, Washington University School of Medicine, St. Louis, MO 63110, USA; 3 Department of Pathology and Immunology, Washington University School of Medicine, St. Louis, MO 63110, USA

**Keywords:** macrophage metabolism, amino acids, muscle repair, inflammation, satellite cells

## Abstract

In this commentary we discuss new findings presented by Shang et al. regarding the role of macrophage-derived glutamine in skeletal muscle repair. Loss-of-function of glutamate dehydrogenase in macrophages led to an upregulation of glutamine synthesis which sustained glutamine levels in muscle tissue and facilitated satellite cell proliferation and differentiation.

Macrophages are cells of the innate immune system that play a critical role in the regulation of inflammatory responses. Tissue resident macrophages contribute to organ development and homeostasis, whereas monocyte-derived macrophages are recruited upon tissue injury and coordinate tissue inflammation and repair. Up until recently, the inflammatory vs. reparative capacity of macrophages was often referred to as M1 or M2 activation states. However, it is now recognized that this terminology fails to describe the true diversity and plasticity of macrophage subsets in vivo [1]. Irrespective, it is well accepted that macrophages can promote tissue repair trough the clearance of dead cells, induction of angiogenesis, and regulation of matrix remodeling [2]. However, based on tissue location and mode of injury the mechanisms by which macrophages affect tissue repair may vary. Therefore, understanding the precise mechanisms utilized by these phagocytes to improve healing is of critical importance.

Over the past decade it has been appreciated that macrophage cellular metabolism often dictates cell activation and effector functions. As an example, inflammatory macrophages are biased towards glycolytic metabolism whereas macrophages with reparative phenotypes tend to rely upon mitochondrial fatty acid oxidation [3,4]. However, it has become clear that this simple model of linking metabolic substrate use to effector phenotype is more complex than previously appreciated [5]. In addition, although the role of amino acid (AA) metabolism in macrophages is gaining more attention, relatively little is known about AAs in macrophage activation states. That being said, the AA glutamine has long been known to influence immune cell activation and polarization and it is for this reason that most cell culture media contains an excess of glutamine. However, in the *in vivo* setting the availability and utilization of glutamine by macrophages in homeostasis and disease is less well understood.

Skeletal muscle injury occurs in cases of trauma, muscular dystrophy, drug toxicity and aging. Macrophages contribute to skeletal muscle regeneration via several mechanisms including the release of cytokines that promote repair, such as IL-6 and TGFβ, and growth factors, including IGF-1, that can stimulate expansion of the muscle stem cells [6–8]. In an elegant recent study, Shang et.al. investigate the role of metabolites as mediators of crosstalk between macrophages and muscle satellite cells, an area which had not previously been explored. The authors describe a novel mechanism linking macrophage glutamine metabolism to muscle repair [9]. The authors used both cardiotoxin and femoral artery ligation models of skeletal muscle injury and first demonstrated that mice with a macrophage-specific knockout (KO) of glutamate dehydrogenase (*Glud1*), GLUD1 KO, had improved resolution of tissue damage and earlier restoration of functional capacity compared to wild type (WT) mice. This occurred as a consequence of enhanced proliferation of muscle satellite cells. Thus, perturbing macrophage glutamine metabolism enhanced muscle repair and regeneration.

Intriguingly, macrophage recruitment and wound healing/angiogenic capacity were similar between the genotypes. Therefore, to understand the mechanism of this phenotype the authors performed metabolic phenotyping of GLUD1 KO macrophages. As GLUD1 catalyzes the conversion of glutamate to α-ketoglutarate for entry into the tricarboxylic acid (TCA) cycle it was not surprising that KO macrophages had a ~75% reduction in glutamine oxidation capacity. Intriguingly, the authors also demonstrated that macrophage glutamine production increased with the loss of GLUD1 and this was associated with an upregulation of the enzyme glutamine synthase (GS). Macrophage-specific KO of GS in GLUD1KO mice prevented the enhanced proliferation of muscle satellite cells that occurred with injury. Thus, loss of GLUD1 in macrophages promoted muscle regeneration via a GS-dependent mechanism.

To understand the potential relevance of enhanced glutamine production to the crosstalk between macrophages and skeletal muscle cells the authors used an in vitro co-culture system. Glutamine is known to be important for myoblast proliferation. When WT macrophages were cultured with myoblasts in glutamine rich media the growth of myoblasts was diminished to levels observed under glutamine-restricted conditions. In contrast, this did not occur when myoblasts were cultured with GLUD1 KO macrophages irrespective of the glutamine quantity added to the media. This data suggested that under normal conditions macrophages take up extracellular glutamine and thereby reduce the amount of this AA that is available for use by myoblasts. In line with this observation, glutamine concentrations decreased in the muscle interstitial fluid following injury in WT mice and this drop did not occur in macrophage GLUD1 KO mice. As such, macrophages appear to compete with satellite cells for glutamine following muscle injury, influencing muscle regeneration.

These findings suggested a model whereby glutamine release from GLUD1 KO macrophages enhanced glutamine availability and fueled muscle satellite cell expansion. To explore this possibility in more detail, the authors knocked out the primary receptor involved in glutamine uptake, SLC1A5, in satellite cells in vitro and in vivo using a CRISPR-Cas9 approach. When satellite cell glutamine uptake was inhibited, the beneficial phenotype observed in macrophage GLUD1 KO mice was lost, confirming that glutamine released from macrophages was driving muscle regeneration. Similar results were observed in GLUD1 KO mice treated with the SLC1A5 inhibitor g-l-glutamyl-*p*-nitroanilide (GPNA). Together these findings confirm that the salutary effects of GLUD1 deficiency in macrophages is dependent on glutamine delivery to satellite cells. Interestingly, the authors also confirmed a protective effect of macrophage GLUD1 deficiency on preservation of muscle mass with aging, indicating possible applications of this concept outside of acute injury.

Despite the elegant mechanistic work performed by the authors, the question remained as to whether this pathway could be translated into therapeutics. Therefore, the investigators treated mice with the GLUD1 inhibitor R162 after muscle injury. Inhibition of this enzyme also improved muscle regeneration and satellite cell proliferation. Moreover, in aged mice R162 treatment for one month improved muscle mass and exercise capacity. Thus, pharmacologic strategies targeting GLUD1 have promise for the treatment of acute and chronic muscle injury.

The study by Shang et.al. is an exciting addition to the field of immunometabolism. The authors likely anticipated that disrupting glutamine oxidation in macrophages would have a direct effect on the macrophage polarization and thereby alter the injury response. Instead, they uncover a novel pathway whereby the release of glutamine from macrophages into the muscle microenvironment drove regeneration and healing. Equally as exciting, this study provides compelling evidence that this pathway could be exploited for therapeutic purposes (Figure 1).

## Figures and Tables

**Figure 1.**
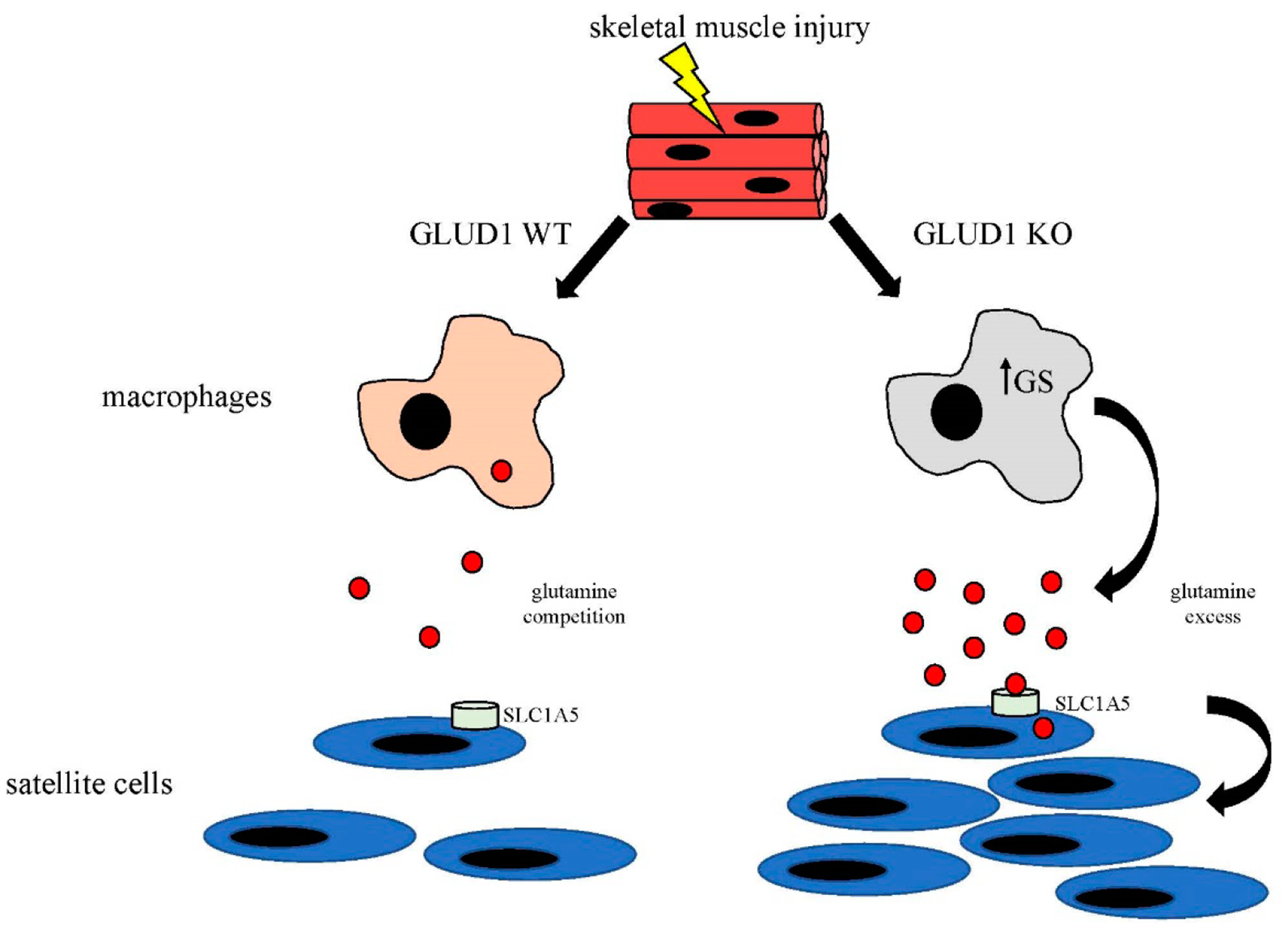
Schematic illustrating the key findings of Shang et. al. study. In response to skeletal muscle injury macrophages enter the tissue and glutamine levels drop. In WT mice, macrophages compete with satellite cells (SC) for glutamine limiting the amount that is available to drive SC proliferation. In contrast, GLUD1 KO macrophages upregulate glutamine synthesis (GS) which leads to release of glutamine into the microenvironment. The glutamine enters SC via the receptor SLC1A5 and promotes SC proliferation, accelerating muscle regeneration.
